# Patterns of social relationships among community-dwelling older adults in Japan: latent class analysis

**DOI:** 10.1186/s12877-022-02748-7

**Published:** 2022-01-25

**Authors:** Kumi Watanabe Miura, Takuya Sekiguchi, Mihoko Otake-Matsuura, Yuko Sawada, Emiko Tanaka, Taeko Watanabe, Etsuko Tomisaki, Rika Okumura, Yuriko Kawasaki, Sumio Ito, Tokie Anme

**Affiliations:** 1 Japan Society for the Promotion of Science (JSPS), Kojimachi, Chiyoda-ku, Tokyo, 102-0083 Japan; 2 RIKEN Center for Advanced Intelligence Project, Nihonbashi, Tokyo, 103-0027 Japan; 3 Morinomiya University of Medical Sciences, Suminoe-ku, Osaka, 559-8611 Japan; 4 Musashino University, Ariake, Koto-ku, Tokyo, 135-8181 Japan; 5 College of Nursing and Nutrition, Shukutoku University, Nitona-cho, Chiba, 260-8703 Japan; 6 Faculty of Nursing and Medical Care, Keio University, Shinanomachi, Tokyo, 160-0016 Japan; 7 Department of Public Welfare, Tobishima, Aichi, 490-1434 Japan; 8 Faculty of Medicine, University of Tsukuba, 1-1-1 Tennodai, Tsukuba, Ibaraki, 305-8575 Japan

**Keywords:** Social relationships, Social isolation, Social support, Older adults, Aging

## Abstract

**Background:**

Social relationships may be the key to successful aging among older adults. However, little is known about the variability of social relationships among community-dwelling older people. This study aimed to describe the patterns of social relationships and examine the differences in sociodemographic characteristics and mental and physical health status among these patterns.

**Methods:**

We obtained the data from a questionnaire survey in 2017 for older adults aged 65 and above who lived in a suburban area in Japan. The Index of Social Interaction (ISI) was used to evaluate social relationships. The final sample comprised 964 people who were independently mobile and answered at least one item of the ISI. To clarify the patterns of social relationships, latent class analysis was performed with five subscales of ISI treated as indicator variables. Multinomial logistic regression was conducted to examine the factors associated with the patterns of social relationships.

**Results:**

The patterns of social relationships were classified into three classes: “Active” (73.6%), “Socially isolated” (14.7%), and “Less motivated” (11.7%). Persons who had depressive symptoms were more likely to be allocated to the “Socially isolated” (Odds Ratio [OR] 1.80, 95% Confidence Interval [CI] 1.13–2.86) or the “Less motivated” groups (OR 1.69, 95% CI 1.00–2.85) compared to the “Active” group. In addition, men (OR 1.72, 95% CI 1.07–2.76) and those living alone (OR 3.07, 95% CI 1.43–6.61) were more likely to be allocated to the “Socially isolated” group. Moreover, those who were dependent, according to the instrumental activities and daily living functions, were more likely to be assigned to the “Socially isolated” (OR 2.19, 95% CI 1.21–3.97) or “Less motivated” (OR 6.29, 95% CI 3.47–11.39) groups.

**Conclusion:**

This study revealed the patterns of social relationships in older adults and suggested that there may be variations of social relationships among community dwellers. The results also indicated the necessity of assessing individual patterns of social relationships and devising strategies for each pattern in public health practice.

**Supplementary Information:**

The online version contains supplementary material available at 10.1186/s12877-022-02748-7.

## Introduction

Social relationships are the key to successful aging in old age. Connecting with friends, family, and society may give life meaning [1], contribute to happiness [2], satisfaction with life [3], and enhance an individual’s emotional well-being [4]. In addition to contributing to emotional well-being, social relationships have been described in several studies as being related to mental and physical health outcomes using various definitions and terms. For example, many studies found that the association between social relationships and mental health outcomes has led to the onset of depression [5–7], while another study reported that being a member of social groups protects individuals against the onset of depression, alleviates symptoms of depression, and prevents depression relapse [8]. In terms of physical health, there may be an association between poor social relationships and all-cause mortality [9–11], cancer mortality [12, 13], and declining physical function [14, 15]. Further, poor social relationships were also associated with cognitive health, such as the onset of dementia [16, 17]. A review by Cohen [18] suggested that social relationships may affect an individual’s health through the mechanism of stress buffering and health behavior modification. Hence, maintaining social relationships has great significance for the maintenance of good health and well-being in public health practices among older people.

Based on evidence from empirical studies, social relationship interventions have been conducted in several studies [19, 20]. Previous studies in Japan reported that interventions based on community activity promotion might decrease the risk of long-term care [21] and declining cognitive functioning [22] among community-dwelling older adults. As awareness of the importance of social relationships increases, accumulating empirical knowledge for enhancing social relationships is critical in public health practices. Further, interventions tailored to target pre-identified barriers and characteristics are likely to improve professional practice [23]. Understanding the profiles of social relationships is expected to be significantly effective even in social relationships interventions.

Recently, several studies have reported attempts to classify social participation patterns. For example, a study in Switzerland identified four unique social participation patterns of older adults based on the type of activities that individuals engage in: “relatively inactive,” “relatively active” (informal activities), “relatively active” (formal activities), and “highly active” [24]. Similarly, Ukawa et al. [25] identified activity types such as “sports groups/clubs and hobby groups,” and “political groups/organizations and industry/trade associations” as social participation patterns, and also found that the risk of long-term care outcomes differed by patterns among the Japanese older adult population. These studies indicate the possibility of variability of individual social patterns. People are exposed to various social environments in their daily lives, and understanding the diverse patterns of social relationships and the factors associated with them among older adults in the community can have important consequences for establishing basic knowledge for health practices. However, there is still a paucity of studies on social relationship patterns of older adults focusing on multiple aspects of social relationships as previous studies have focused on specific elements. Further, it is unclear how demographic factors are associated with these patterns.

Therefore, this study aims to 1) identify the patterns of various aspects of social relationships in daily life using latent class analysis (LCA) and 2) examine the differences in the patterns according to people’s socio-demographic characteristics and mental and physical health status. Our approach that defines social relationships by multiple aspects rather than a specific element may describe the diversity of social relationships and reveal the subtypes of low social relationships, which have previously been recognized as a single group. Understanding the diverse patterns and its associated factors may provide insights for the development of public health interventions and strategies.

## Materials and methods

### Study design and the participants

This study employed a cross sectional study design and used data from the “Community empowerment and care for well-being and healthy longevity study (CEC study).” The study involves a questionnaire survey conducted every 2–3 years for all the residents living in a suburban area (total population = 4539) in central Japan, and investigates the factors related to well-being and longevity. The questionnaires were distributed using “the drop-off/pick-up” method. Accordingly, municipality-assigned volunteers visited all residents living in the study setting and distributed the questionnaires. After 2 weeks, volunteers revisited the participants to collect the completed questionnaires. We used the data from the 2017 wave (April 2017) for this study. The two inclusion criteria for participants of this study were older adults aged over 65 years who were independent and mobile. Of a total of 1085 participants, who responded to the 2017 wave survey (response rate: 85.1%), 964 participants who met inclusion criteria and answered at least one item of the ISI were included in the final sample.

### Social relationships

The Index of Social Interaction (ISI), which was developed to measure various aspects of social relationships in daily settings, showed high validity and reliability (Cronbach alpha 0.78) among Japanese community-dwelling older adults [26], and was therefore used in this study to provide the indicator variables. The ISI consists of 18 items and five subscales, each rated on a 4- or 5-point scale: 1) “independence,” which measures social control involving feelings of obligation or responsibility toward a healthier lifestyle; 2) “social curiosity,” which evaluates intellectual activities such as reading (e.g., newspapers, books, or magazines), trying to use new equipment, and having a hobby; 3) “interaction,” which includes social networking such as family communication, non-family communication, and interactions with non-family persons; 4) “participation,” which measures social participation such as participation in social groups, neighborhood-activities, and taking an active role in society; and 5) “feeling safe,” which evaluates social support from the receipt of counseling and having someone to provide support in an emergency. One point was given for each item with a positive response, and zero points were given for a negative response, and each subscale score was calculated by summing up the item scores. The distribution exhibited high skewness (Supplement 1), and accordingly, the ISI subscale scores were dichotomized into the “high (> perfect score)” and “low (< perfect score)” groups to conduct the LCA analysis.

### Covariates

Age, sex, family structure, subjective economic status, the instrumental activities and daily living (IADL) functions, disease status, and depressive symptoms were introduced as covariates. Information on the covariates was obtained from the questionnaires.

While age was treated as a continuous variable, family structure was categorized as living alone or other. Subjective economic status was obtained by asking one question: How severe do you feel your current living economic situation is? Participants who answered, “Very severe” and “Severe” were defined as the “poor” group, and other answers such as “Average” and “Good” were defined as the “better-off” group. The IADL variable was used with the subscale of IADL in the Tokyo Metropolitan Institute of Gerontology Index of Competence [27]. The items had three options for each question according to the guidelines of the Ministry of Health, Labour and Welfare in Japan [28]. The IADL scores ranged from 0 to 5 in which a score of 5 was defined as independent and any score between 0 and 4 was defined as dependent. The disease status (heart disease, diabetes, and musculoskeletal disorder) of the participants was defined based on their answers. Depressive symptoms were screened and assessed through two questions that asked about depressed moods [29]. If a participant answered “yes” to at least one question, they were defined as having depressive symptoms.

### Statistical analysis

In this study, we determined (1) the pattern of social relationships and (2) the association between demographic factors and the patterns of social relationships.

First, LCA was conducted to determine the patterns of social relationships. LCA is a method that can be useful for clustering subgroups of latent classes [30]. In this study, five subscales of social relationships in the ISI were used as observed indicators in the LCA. The number of latent classes was comprehensively determined by comparing robust indicators including the Bayesian Information Criterion (BIC), the adjusted Bayesian Information Criterion (aBIC), the Akaike Information Criterion (AIC) and the consistent Akaike Information Criterion (CAIC) among each of the models. Entropy was used to assess the accuracy of clustering. We used the full information maximum likelihood estimation to deal with missing values in the ISI scale. Participants with data missing in all the items of ISI were removed.

Second, participants were assigned to their most likely latent class after the best fit model was selected. A multinomial logistic regression model was subsequently computed to examine the association between the characteristics and the social relationship subtypes obtained. To deal with missing data in the multinomial logistic regression model, we used multiple imputation by the fully conditional specification method for covariates. We created 50 imputed data sets and integrated each result of the analysis. The level of significance was set at 0.05. All statistical tests were performed using the SAS 9.4 version.

## Results

### Model fitting

Table 1 shows the model fit indicators of the LCA. We adopted the three-class model based on the lowest AIC (54.22), BIC (137.03), and aBIC (83.04), and interpretability. The three-class model showed a high entropy value (0.70), which justified conducting the regression analysis after assigning participants to their most likely latent class.

**Table 1:** Model comparison of latent class analysis fit in social relationship patterns

No. classes	AIC	BIC	CAIC	aBIC	Entropy
1	337.65	362.01	367.01	346.13	1.00
2	84.65	138.23	149.23	103.29	0.66
3	54.22	137.03	154.03	83.04	0.70
4	57.22	169.26	192.26	96.21	0.60
5	63.6	204.87	233.87	112.76	0.68

*Note*: *AIC* Akaike Information Criterion, *BIC* Bayesian Information Criterion, *CAIC* Consistent Akaike Information Criterion, *aBIC* Adjusted Bayesian Information Criterion

Supplementary Tables 1 and 2 present the descriptive statistics and correlation matrix for ISI scores. Subsequently, the independence and social curiosity subscales of ISI reported medium correlation.

### Patterns of social relationships

The conditional probabilities for each class are listed in Table 2. The three classes were identified as “Active” (73.6%), “Socially isolated” (14.7%), and “Less motivated” (11.7%). Supplementary Table 3 presents the number of missing data among the three latent classes; 65.7% of participants have no missing data and 23.5% have missing data on only 1 ISI subscale. This means that 89.2% of participants have missing data on 0–1 ISI subscales. Furthermore, only 0.6% of the participants have missing data in four subscales, which suggests that there were few cases where participants with few responses were inaccurately assigned to classes due to a lack of information.

**Table 2:** Conditional item response probability of social relationships

	Independence	Social curiosity	Interaction with others	Social participation	Feeling safe
Class1: Active (73.6%)	0.998	0.788	0.972	0.689	0.989
Class 2: Socially Isolated (14.7%)	0.998	0.696	0.555	0.151	0.680
Class 3: Less motivated (11.7%)	0.256	0.092	0.664	0.221	0.706

The first class consisted of people with the comparatively highest conditional probabilities for all subscales that reflect highly social relationships. They were likely to engage in all aspects of social activities in their daily lives. This class was labeled as the “Active” class. The second class had the lowest conditional probability for interaction with others, social participation, and the feeling of safety. This class was labeled as the “Socially isolated” class. The subjects in the third class showed relatively lower conditional probability of independence and social curiosity. This class was labeled as “Less motivated.”

### The association between demographic characteristics and class membership of social relationships

Table 3 shows demographic characteristics and ISI indicators within the three latent classes of social relationships. The mean age for “Active,” “Socially isolated,” and “Less motivated” groups was 73.8 (± 6.85), 73.6 (± 7.59), and 78.3 (± 8.26), respectively. Moreover, males accounted for 43.1, 55.1, and 42.9% of the “Active,” “Socially isolated,” and “Less motivated” group, respectively. For ISI subscales, the “Less motivated” group reported relatively smaller means on the independence (2.50 ± 0.74) and social curiosity subscales (3.58 ± 0.67) than the “Active” and “Socially isolated” groups. Additionally, the “Socially isolated” group reported smaller means on the interaction (2.10 ± 0.74) and participation subscales (2.69 ± 0.73) than the “Active” and “Socially isolated” groups.

**Table 3:** Demographic factors within three latent classes of social relationships

	Active		Socially Isolated		Less motivated	
	n	%	n	%	n	%
Age						
Mean (±SD)	73.8	(± 6.85)	73.6	(± 7.59)	78.3	(± 8.26)
Missing	0		0		0	
Sex						
Male	337	43.1%	54	55.1%	36	42.9%
Female	395	50.5%	36	36.7%	45	53.6%
Missing	50	6.4%	8	8.2%	3	3.6%
Subjective economic status						
Better-off	578	73.9%	66	67.3%	57	67.9%
Poor	144	18.4%	23	23.5%	22	26.2%
Missing	60	7.7%	9	9.2%	5	6.0%
Chronic disease						
Heart disease	52	6.6%	8	8.2%	7	8.3%
Diabetes	114	14.6%	13	13.3%	16	19.0%
Musculoskeletal disorder	72	9.2%	9	9.2%	8	9.5%
Missing	0	0.0%	0	0.0%	0	0.0%
IADL						
Independent	621	79.4%	69	70.4%	31	36.9%
Dependent	98	12.5%	21	21.4%	45	53.6%
Missing	63	8.1%	8	8.2%	8	9.5%
Depressive symptoms						
Having	208	26.6%	40	40.8%	33	39.3%
Not having	514	65.7%	46	46.9%	33	39.3%
Missing	60	7.7%	12	12.2%	18	21.4%
Family structure						
Living with someone	663	84.8%	78	79.6%	67	79.8%
Living alone	33	4.2%	11	11.2%	3	3.6%
Missing	86	11.0%	9	9.2%	14	16.7%
ISI Independence						
Mean (±SD)	4.00	(± 0.00)	4.00	(± 0.00)	2.50	(± 0.74)
Missing	38		3		2	
Social curiosity						
Mean (±SD)	4.74	(±0.50)	4.59	(± 0.61)	3.58	(± 0.67)
Missing	174		20		13	
Interaction with others						
Mean (±SD)	2.97	(± 0.19)	2.10	(± 0.72)	2.52	(± 0.75)
Missing	56		2		7	
Participation						
Mean (±SD)	3.60	(± 0.61)	2.69	(± 0.73)	2.77	(± 0.96)
Missing	103		15		20	
Feeling safe						
Mean (±SD)	1.99	(± 0.14)	1.23	(± 0.81)	1.53	(± 0.75)
Missing	15		1		1	

*Note*: *IADL* Instrumental Activities of Daily living, *SD* Standard Deviation

Table 4 shows the results of the crude and adjusted multinomial logistic regression models to examine the differences in demographic characteristics among social relationship patterns by showing which factors were associated with the higher likelihood of belonging to either the “Socially isolated” or the “Less motivated” group than to the reference category, the “Active” group. Persons who had depressive symptoms were more likely to be allocated to the “Socially isolated” or “Less motivated” groups (“Socially isolated”: Odds Ratio [OR] 1.80, 95% Confidence Interval [CI] 1.13–2.86; “Less motivated”: OR 1.69, 95% CI 1.00–2.85) after being controlled by covariates. Males (OR 1.72, 95% CI 1.07–2.76) and those living alone (OR 3.07, 95% CI 1.43–6.61) were more likely to be allocated to the “Socially isolated” group. In addition, those who were dependent according to the IADL function were more likely to be assigned to the “Less motivated” and “Socially isolated” groups (“Socially isolated”: OR 2.19, 95% CI 1.21–3.97; “Less motivated”: OR 6.29, 95% CI 3.47–11.39). Similarly, we developed models using the “Less motivated” group as the reference category to examine the differences in the demographic characteristics of the “Socially isolated” and “Less motivated” groups (See Supplementary Table 4). Those who were younger (OR 0.95, 95% CI 0.91–0.99), males (OR 2.01, 95% CI 1.05–3.83), and independent in IADL (OR 0.35, 95% CI 0.16–0.76) were more likely to be allocated to the “Socially isolated” group.

**Table 4:** Multinomial logistic regression analysis for class memberships of social relationships

Class 2: Socially Isolated (ref = Class 1: Active)	Crude Odds	95% CI	Adjusted Odds	95% CI
Age (Continuous)	1.08***	1.05–1.11	0.98	0.95–1.01
Sex = Male (ref. female)	1.69*	1.08–2.65	1.72*	1.07–2.76
Subjective economic status = Poor (ref. Better-off)	1.38	0.83 2.29	1.11	0.65–1.88
Chronic disease = Having (ref. Not having)				
Heart disease	1.25	0.57–2.71	1.05	0.47–2.37
Diabetes	0.90	0.48–1.66	0.82	0.44–1.55
Musculoskeletal disorder	1.00	0.48–2.06	0.99	0.46–2.15
IADL = Dependent (ref. Independent)	1.91*	1.14–3.22	2.19**	1.21–3.97
Depressive symptoms = Having (ref. Not having)	1.99**	1.28–3.11	1.80*	1.13–2.86
Living status = Living alone (ref = Living with someone)	2.59**	1.27–5.31	3.07**	1.43–6.61
Class 3: Less motivated (ref = Class 1: Active)	Crude Odds	95% CI	Adjusted Odds	95% CI
Age (Continuous)	1.00	0.97–1.03	1.03	0.99–1.06
Sex = Male (ref. female)	0.93	0.59–1.47	0.86	0.52–1.42
Subjective economic status = Poor (ref. Better-off)	1.51	0.89–2.54	1.32	0.75–2.35
Chronic disease = Having (ref. Not having)				
Heart disease	1.28	0.56–2.91	0.88	0.36–2.13
Diabetes	1.38	0.77–2.46	1.32	0.71–2.47
Musculoskeletal disorder	1.04	0.48–2.24	0.83	0.36–1.91
IADL = Dependent (ref. Independent)	8.00***	4.90–13.07	6.29***	3.47–11.39
Depressive symptoms = Having (ref. Not having)	2.10**	1.30–3.41	1.69*	1.00–2.85
Living status = Living alone (ref = Living with someone)	0.92	0.28–3.07	1.41	0.39–5.09

*Note*: Ref: “Active,” * *P* < 0.05; ** *P* < 0.01; *** *P* < 0.001, *IADL* Instrumental Activities of Daily living, *CI* Confidence Interval

## Discussion

Despite the importance of social relationships for older people, the variability of social relationships has not been well understood; in addition, limited literature is available focusing on specific aspects of social relationships such as social participation activities. Thus, this study extracted the latent patterns across multiple aspects of social relationships among Japanese community-dwelling older adults.

We extracted three social relationships patterns: “Active,” “Socially isolated,” and “Less motivated” groups. This study not only showed that there are variations of social relationships among community dwellers, which is consistent with previous studies that described social activities patterns [24, 25, 31, 32], but also found that inactive social relationships can be classified into two subtypes, which were previously seen as one. Interestingly, the comparison of the “Socially isolated” and “Less motivated” groups did not indicate a simple relationship in which one group’s response probability was higher than that of the other in any domain of social relationships. This result meant that the two groups have different features in each domain, and that the social relationships between the two groups are qualitatively different, rather than simply expressing high and low. This finding may be useful in devising effective strategies for each pattern in public health practice.

We also investigated the differences in the factors associated with the higher probability of being allocated to the “Socially isolated” or “Less motivated” groups compared with the “Active” group. First, depressive symptoms were a common factor associated with the likelihood of being allocated to the “Socially isolated” or “Less motivated” groups. This result highlights the importance of improving depression interventions in both patterns of inactive social relationships. Once we consider these two groups as relatively inactive groups, this result may be consistent with previous studies that showed the association between poor social relationships and depressive symptoms. However, previous studies have reported a bidirectional relationship between social relationships and depression: Poor social relationships is associated with poor mental health outcomes [5–8], and depression may further reduce social involvement [33, 34]. The two inactive groups in the present study may be differently associated with depression both in terms of direction of causality and the associated effect sizes. Therefore, future longitudinal studies that clarify similarities and differences between the two groups and causality in more detail should be conducted to provide fruitful insights to improve the efficacy of interventions.

In addition, people who were dependent in their IADL functions were more likely to be allocated to the “Less motivated” or “Socially isolated” group. Furthermore, compared to the “Less motivated” group, the “Socially isolated” group showed significantly lower probability of dependency in their IADL function. While older adults experience functional decline and various aspects of loss with aging, social relationships are also said to reduce with time [35]. Another study also reported that social participation, such as poor participation in hobby clubs, senior citizen clubs, and volunteer groups was associated with future occurrence of IADL decline [36]. Considering that social frailty may precede physical frailty [37], it may be said that the “Less motivated” group is a group in which the beginnings of functional decline occur. Secondary prophylactic interventions may be essential in the “Less motivated” group to prevent further functional decline.

Being male and living alone were associated with being allocated to the “Socially isolated” group. Our result is consistent with a previous study that reported the association of demographic factors such as sex, educational attainment, economic, and marital status with social isolation [38]. This study found that an understanding of the subject’s background, such as cohabitation, might be desirable as a screening and intervention approach to the “Socially isolated” group. However, despite the reports about the impact on economic status [38, 39], it could not be confirmed using our model. One possible reason is that our study evaluated subjective economic status where participants were asked how they felt about their living economic status rather than an objective indicator. The gap between the subjective and actual situations could affect our results. Further, the effect of the other confounding factors also could be such that economic status may be associated with the transition to living alone [40] while living arrangement was associated with the “Socially isolated” group in this study. Although this study could not confirm the independent effect of subjective economic status, detailed further examination is necessary to consider complex background risk factors for developing interventions.

The limitations of this study should be noted. First, it was conducted in a limited area, which reduces the generalizability of the results. Additionally, the lack of power owing to the relatively small sample size may have affected the results. The small sample size of “Socially isolated” and “Less motivated” groups may reduce the power to detect the association between covariates and the likelihood of belonging to those groups. Further research should employ larger sample sizes to provide further and more robust evidence of the results. The current results may also be affected by a selection bias, given a possible association between the ISI subscales and actively participating for various events including our survey. Therefore, we cannot rule out the possibility that sizes of “Socially isolated” and “Less motivated” groups may be underestimated. In addition, people who did not participate in the survey might have belonged to another latent class apart from those identified in our analysis, which may be overcome by exploratory qualitative research such as interviews. Second, there may be other confounding factors like educational attainment that were not included in the data used in this study. Investigating possible confounding variables in future research is necessary. Third, we used data that was obtained before the COVID-19 pandemic. The pandemic has significantly changed people’s way of life and social relationships. In the future, it would be useful to investigate the kind of changes that occurred in social relationships during and after the pandemic. Based on these limitations, further studies are expected to be conducted to develop this research topic.

## Conclusion

This study derived three social relationship patterns—“Active,” “Socially isolated,” and “Less motivated” groups, and investigated differences in socio-demographic characteristics and mental and physical health status in those patterns. Inactive social relationships could be classified into two subtypes, and the characteristics of participants may differ by patterns of social relationships. Our work suggests the necessity of assessing individual’s patterns of social relationships and creating strategies separately for each pattern, in the practice of public health: (1) intervention in mental health for both “Socially isolated” and “Less motivated” groups is essential as a comprehensive intervention policy for older adults, (2) in addition to mental health care, secondary prevention care for functions such as IADL may be necessary for “Less motivated” group, which may be declining social functioning, (3) since “Socially isolated” group tend to have demographic risks, such as living arrangement, understanding the background of the target is essential when intervention or screening is deemed necessary. Our finding is expected to contribute to the development of more effective interventions in the future.

## Supplementary Information

**Additional file 1: Supplementary Table 1.** Descriptive statistics of ISI scores.

**Additional file 2: Supplementary Table 2.** Correlation matrix for the five ISI subscales.

**Additional file 3: Supplementary Table 3.** The number of missing data among three latent classes.

**Additional file 4: Supplementary Table 4.** Multinomial logistic regression analysis for class memberships of social relationships compared to “Less motivated” group.

## Data Availability

The datasets are available from the corresponding author on reasonable request and if permission from the municipality of study area is granted.

## Abbreviations

LCA: Latent class analysis
CEC study: Community empowerment and care for well-being and healthy longevity study
ISI: Index of Social Interaction
IADL: Instrumental activities and daily living
BIC: Bayesian Information Criterion
aBIC: Adjusted Bayesian Information Criterion
AIC: Akaike Information Criterion
CAIC: Consistent Akaike Information Criterion
