# Supplementary material for: Patterns of social relationships among community-dwelling older adults in Japan: latent class analysis
Source: BMC Geriatr. 2022 Jan 25;22:75. doi: 10.1186/s12877-022-02748-7 (PMC8786623; doi:10.1186/s12877-022-02748-7)
Supplement: Supplementary file 1 — Additional file 1: Supplementary Table 1. Descriptive statistics of ISI scores. [file 12877_2022_2748_MOESM1_ESM.docx]

Supplementary Table 1. Descriptive statistics of ISI scores

|  | Score | n | % |
| --- | --- | --- | --- |
| ISI Independence | Mean (±SD) = 3.87 (± 0.48), Skewness = -4.23 | | |
|  | 0 | 1 | 0.1% |
|  | 1 | 9 | 0.9% |
|  | 2 | 20 | 2.1% |
|  | 3 | 52 | 5.4% |
|  | 4 | 839 | 87.0% |
|  | Missing | 43 | 4.5% |
| Social curiosity | Mean (±SD) = 4.61 (± 0.63), Skewness = -1.42, | | |
|  | 0 | 0 | 0.0% |
|  | 1 | 0 | 0.0% |
|  | 2 | 1 | 0.1% |
|  | 3 | 56 | 5.8% |
|  | 4 | 178 | 18.5% |
|  | 5 | 522 | 54.1% |
|  | Missing | 207 | 21.5% |
| Interaction | Mean (±SD) = 2.84 (± 0.46), Skewness = -3.30 | | |
|  | 0 | 5 | 0.5% |
|  | 1 | 20 | 2.1% |
|  | 2 | 88 | 9.1% |
|  | 3 | 786 | 81.5% |
|  | Missing | 65 | 6.7% |
| Participation | Mean (±SD) = 3.44 (± 0.73), Skewness ­= -1.31 | | |
|  | 0 | 0 | 0.0% |
|  | 1 | 22 | 2.3% |
|  | 2 | 54 | 5.6% |
|  | 3 | 288 | 29.9% |
|  | 4 | 462 | 47.9% |
|  | Missing | 138 | 14.3% |
| Feeling safe | Mean (±SD) = 1.87 (± 0.44), Skewness = -3.44 | | |
|  | 0 | 39 | 4.0% |
|  | 1 | 46 | 4.8% |
|  | 2 | 862 | 89.4% |
|  | Missing | 17 | 1.8% |
