# Supplementary material for: Patterns of social relationships among community-dwelling older adults in Japan: latent class analysis
Source: BMC Geriatr. 2022 Jan 25;22:75. doi: 10.1186/s12877-022-02748-7 (PMC8786623; doi:10.1186/s12877-022-02748-7)
Supplement: Supplementary file 2 — Additional file 2: Supplementary Table 2. Correlation matrix for the five ISI subscales. [file 12877_2022_2748_MOESM2_ESM.docx]

Supplementary Table 2. Correlation matrix for the five ISI subscales

|  | Social curiosity | Interaction | Participation | Feeling safe |
| --- | --- | --- | --- | --- |
| Independence | 0.471 | 0.198 | 0.236 | 0.237 |
| Social curiosity |  | 0.176 | 0.232 | 0.142 |
| Interaction |  |  | 0.291 | 0.281 |
| Participation |  |  |  | 0.253 |

Note: Spearman’s rank correlation coefficient
