# Supplementary material for: Patterns of social relationships among community-dwelling older adults in Japan: latent class analysis
Source: BMC Geriatr. 2022 Jan 25;22:75. doi: 10.1186/s12877-022-02748-7 (PMC8786623; doi:10.1186/s12877-022-02748-7)
Supplement: Supplementary file 3 — Additional file 3: Supplementary Table 3. The number of missing data among three latent classes. [file 12877_2022_2748_MOESM3_ESM.docx]

Supplementary Table 3. The number of missing data among three latent classes

| No. of   ISI subscales with missing data | Total | Active | Socially Isolated | Less motivated |
| --- | --- | --- | --- | --- |
| 0 | 633 | 518 | 65 | 50 |
|  | 65.7% | 66.2% | 66.3% | 59.5% |
| 1 | 227 | 174 | 27 | 26 |
|  | 23.5% | 22.3% | 27.6% | 31.0% |
| 2 | 75 | 64 | 4 | 7 |
|  | 7.8% | 8.2% | 4.1% | 8.3% |
| 3 | 23 | 20 | 2 | 1 |
|  | 2.4% | 2.6% | 2.0% | 1.2% |
| 4 | 6 | 6 | 0 | 0 |
|  | 0.6% | 0.8% | 0.0% | 0.0% |
