# Supplementary material for: Patterns of social relationships among community-dwelling older adults in Japan: latent class analysis
Source: BMC Geriatr. 2022 Jan 25;22:75. doi: 10.1186/s12877-022-02748-7 (PMC8786623; doi:10.1186/s12877-022-02748-7)
Supplement: Supplementary file 4 — Additional file 4: Supplementary Table 4. Multinomial logistic regression analysis for class memberships of social relationships compared to “Less motivated” group. [file 12877_2022_2748_MOESM4_ESM.docx]

Supplementary Table 4. Multinomial logistic regression analysis for class memberships of social relationships compared to “Less motivated” group

| Class 1: Active (ref = Class 3: Less motivated) | Crude Odds | 95 % CI | | |  | Adjusted Odds | 95 % CI | | |
| --- | --- | --- | --- | --- | --- | --- | --- | --- | --- |
| Age (Continuous) | 0.92*** | 0.90 | - | 0.95 |  | 0.97 | 0.94 | - | 1.01 |
| Sex = Male (ref. female) | 1.08 | 0.68 | - | 1.70 |  | 1.17 | 0.71 | - | 1.92 |
| Subjective economic status = Poor (ref. Better-off) | 0.66 | 0.39 | - | 1.12 |  | 0.76 | 0.43 | - | 1.34 |
| Chronic disease = Having (ref. Not having) |  |  |  |  |  |  |  |  |  |
| Heart disease | 0.78 | 0.34 | - | 1.79 |  | 1.14 | 0.47 | - | 2.77 |
| Diabetes | 0.73 | 0.41 | - | 1.29 |  | 0.76 | 0.40 | - | 1.41 |
| Musculoskeletal disorder | 0.96 | 0.45 | - | 2.08 |  | 1.21 | 0.52 | - | 2.80 |
| IADL = Dependent (ref. Independent) | 0.13*** | 0.08 | - | 0.20 |  | 0.16*** | 0.09 | - | 0.29 |
| Depressive symptoms = Having (ref. Not having) | 0.48** | 0.29 | - | 0.77 |  | 0.59* | 0.35 | - | 1.00 |
| Living status = Living alone (ref = Living with someone) | 1.08 | 0.33 | - | 3.59 |  | 0.71 | 0.20 | - | 2.57 |
| Class 2: Socially isolated (ref = Class 3: Less motivated) | Crude Odds | 95 % CI | | |  | Adjusted Odds | 95 % CI | | |
| Age (Continuous) | 0.92*** | 0.89 | - | 0.96 |  | 0.95* | 0.91 | - | 0.99 |
| Sex = Male (ref. female) | 1.82 | 0.99 | - | 3.34 |  | 2.01* | 1.05 | - | 3.83 |
| Subjective economic status = Poor (ref. Better-off) | 0.92 | 0.47 | - | 1.81 |  | 0.84 | 0.40 | - | 1.72 |
| Chronic disease = Having (ref. Not having) |  |  |  |  |  |  |  |  |  |
| Heart disease | 0.98 | 0.34 | - | 2.82 |  | 1.20 | 0.39 | - | 3.65 |
| Diabetes | 0.65 | 0.29 | - | 1.44 |  | 0.62 | 0.27 | - | 1.43 |
| Musculoskeletal disorder | 0.96 | 0.35 | - | 2.61 |  | 1.21 | 0.42 | - | 3.49 |
| IADL = Dependent (ref. Independent) | 0.24*** | 0.12 | - | 0.46 |  | 0.35** | 0.16 | - | 0.76 |
| Depressive symptoms = Having (ref. Not having) | 0.95 | 0.51 | - | 1.76 |  | 1.07 | 0.55 | - | 2.05 |
| Living status = Living alone (ref = Living with someone) | 2.81 | 0.76 | - | 10.42 |  | 2.18 | 0.54 | - | 8.88 |

Note: Ref: “Less motivated,” * P < 0.05; ** P< 0.01; *** P < 0.001, IADL: Instrumental Activities of Daily living; CI: Confidence Interval
